# The Role of Mesenchymal Stem Cells in Atherosclerosis: Prospects for Therapy via the Modulation of Inflammatory Milieu

**DOI:** 10.3390/jcm8091413

**Published:** 2019-09-08

**Authors:** Armita Mahdavi Gorabi, Maciej Banach, Željko Reiner, Matteo Pirro, Saeideh Hajighasemi, Thomas P. Johnston, Amirhossein Sahebkar

**Affiliations:** 1 Department of Basic and Clinical Research, Tehran Heart Center, Tehran University of Medical Sciences, Tehran 1411713138, Iran; 2 Department of Hypertension, WAM University Hospital in Lodz, Medical University of Lodz, Zeromskiego 113, 90-549 Lodz, Poland; 3 Polish Mother’s Memorial Hospital Research Institute (PMMHRI), 93-338 Lodz, Poland; 4 Department of Internal medicine, University Hospital Center Zagreb, Kišpatićeva 12, Zagreb 1000, Croatia; 5 Unit of Internal Medicine, Angiology and Arteriosclerosis Diseases, Department of Medicine, University of Perugia, 06123 Perugia, Italy; 6 Department of Medical Biotechnology, Faculty of Paramedicine, Qazvin University of Medical Sciences, Qazvin 1531534199, Iran; 7 Division of Pharmacology and Pharmaceutical Sciences, School of Pharmacy, University of Missouri-Kansas City, Kansas City, MO 64110, USA; 8 Biotechnology Research Center, Pharmaceutical Technology Institute, Mashhad University of Medical Sciences, Mashhad 91778-99191, Iran; 9 Neurogenic Inflammation Research Center, Mashhad University of Medical Sciences, Mashhad 91778-99191, Iran; 10 School of Pharmacy, Mashhad University of Medical Sciences, Mashhad 91778-99191, Iran

**Keywords:** atherosclerosis, mesenchymal stem cells, inflammation, cytokines, therapy

## Abstract

Atherosclerosis is a chronic, inflammatory disease that mainly affects the arterial intima. The disease is more prevalent in middle-age and older individuals with one or more cardiovascular risk factors, including dyslipidemia, hypertension, diabetes, smoking, obesity, and others. The beginning and development of atherosclerosis has been associated with several immune components, including infiltration of inflammatory cells, monocyte/macrophage-derived foam cells, and inflammatory cytokines and chemokines. Mesenchymal stem cells (MSCs) originate from several tissue sources of the body and have self-renewal and multipotent differentiation characteristics. They also have immunomodulatory and anti-inflammatory properties. Recently, it was shown that MSCs have a regulatory role in plasma lipid levels. In addition, MSCs have shown to have promising potential in terms of treatment strategies for several diseases, including those with an inflammatory component. In this regard, transplantation of MSCs to patients with atherosclerosis has been proposed as a novel strategy in the treatment of this disease. In this review, we summarize the current advancements regarding MSCs for the treatment of atherosclerosis.

## 1. Introduction

Atherosclerosis is a vascular disease that can progress to the point of occlusion of the arterial lumen and thus, can possibly cause several critical complications, such as coronary artery disease and myocardial infarction [1,2,3]. Among the risk factors for atherosclerosis, hypertension, dyslipidemia, diabetes, obesity, and smoking have particularly detrimental impacts [4,5]. Atherosclerosis is a leading cause of mortality around the world despite a lot of efforts in the management and treatment of cardiovascular risk factors [6]. The pathogenesis of atherosclerosis is complex and has been primarily attributed to lipoprotein accumulation in the subendothelial space, activation or dysfunction of endothelial cells (ECs), infiltration of monocytes and macrophages, and their subsequent transformation into foam cells, which is triggered by oxidized low-density lipoproteins (ox-LDLs) [7,8,9,10]. Innate immune components participate in cholesterol uptake via pattern recognition receptors (PRRs), but they also participate in EC dysfunction and the development of foam cells [11]. Moreover, for participants in adaptive immunity, primarily T cells, which are locally activated in the intima, initiate an inflammatory response and, therefore, participate in further worsening of the development of atherosclerotic lesions [12,13]. As a consequence, therapeutic strategies aimed at immunosuppression and controlling the inflammatory response may be promising in the management of atherosclerotic complications [14,15]. In this context, it was postulated that not all the benefits of statins, the mainstay of treatment in patients with atherosclerotic cardiovascular disease, can be attributed to the cholesterol-lowering action of these drugs. Statins also possess pleiotropic effects [16,17,18,19,20,21], particularly anti-inflammatory properties [22,23], which contributes to the reduction of cardiovascular events. A more relevant piece of evidence was provided by the CANTOS trial, in which canakinumab, a monoclonal antibody against interleukin (IL)-1β, was efficacious in lowering cardiovascular events in a lipid-independent manner [24].

Recent directions of research have been concentrated on cell-based therapies. Mesenchymal stem cells (MSCs) are regarded as the first choice of stem cells for use in regenerative medicine [25]. MSCs have been obtained from various tissues, including the brain, heart, and kidney [26,27]. The main reasons that MSCs are candidates for cell therapy is their well-established ability to differentiate into different cell types and their possibility of in vitro expansion. After in vitro intervention and re-implantation in the body, MSCs develop the ability to suppress various components of the immune system and its response.

Recently, it was reported that MSCs can function as protectors against inflammation [28,29]. Thus, for instance, the adoptive transfer of MSCs was proposed as a novel therapeutic tool to treat atherosclerosis due to their ability to modulate and attenuate the inflammation, which is associated with atherosclerosis [30]. A better understanding of the functions of MSCs in animal models of atherosclerosis, as well as of the mechanisms underlying their therapeutic potential, should encourage further investigation of MSCs in well-designed clinical trials and identification of their possible application in clinical practice.

## 2. Characteristics of MSCs

MSCs were first recognized in the stromal matrix of the bone marrow [31,32]. The understanding of the characteristics and localization of MSCs in the human body is still incomplete. In addition to bone marrow, MSCs have also been found in tissues such as cord blood, placenta, amniotic fluid, skeletal muscle, heart, synovial tissue, adipose tissue, pancreas, and circulating blood [33,34]. It has been supposed that all human body organs, including connective tissues, also contain MSCs [35]. From an embryological perspective, MSCs are considered to be primitive cells that originate from the mesodermal germ layer. They are regarded as progenitors cells that can differentiate and develop into several tissue types, such as connective tissue, skeletal muscle cells, and cells related to the vascular system. MSCs can also develop into mesodermal cell lineages, including bone, cartilage, and fat. In fact, they can also develop into endodermic and neuroectodermic lineages [36,37]. Due to their suggested potential in self-renewal and differentiation, bone marrow-derived stromal cells were first named as stem cells but were later called MSCs [38], which has caused some problems with this inconclusive nomenclature [39]. Among stem cells, MSCs have become the first and best choice for regenerative medicine because they are easy to obtain and harvest, they exhibit rapid ex vivo proliferation, and they have the potential for autologous transplantation [28,40]. MSC involvement in the etiopathogenesis of immunology-based diseases and their ability to modulate the immune response make MSCs an intriguing cell line of which its therapeutic applications are progressively increasing with the idea to control inflammation-related disorders [41,42,43].

## 3. Origin of MSCs

The extraction of MSCs is less invasive than other stem cell types [26]. MSCs have plasticity for development into lineages of different tissue types, either within or across germ lines [44], and bone marrow-derived MSC (BM-MSCs) have the highest level of lineage plasticity [45]. Most preclinical trials have been performed using BM-MSCs for atherosclerosis treatment. However, bone marrow might not represent the most appropriate source for therapeutic applications. This is because the harvesting of bone marrow requires invasive procedures and it yields a low supply of cells. Furthermore, the count, differentiation capacity, and lifespan of BM-MSCs decreases with patient age [46,47]. Two other alternative sources for harvesting MSCs include adipose tissue and umbilical cord (UC) blood, which have drawn significant attention in the last several decades [48,49]. MSCs obtained from adipose tissue have become attractive since adipose tissue can be collected easily and it provides a rich supply of cells with satisfactory proliferative capacity in vitro [50]. MSCs obtained from the adipose tissue and bone marrow are similar with respect to their ability for expansion and differentiation, as well as their immunophenotypes [51], but UC-derived MSCs (UC-MSCs) were just once used in experiments concerning atherosclerosis. MSCs isolated from dental pulp have not been used for atherosclerosis experiments [52]. Also, UC blood and Wharton’s jelly are rich sources of MSCs [53,54].

## 4. Colonization and Migration of MSCs

The initial key step for MSCs targeting other tissues and organs is their mobilization from the source organs. It has been shown that endogenous MSCs can be mobilized from the source tissues to the peripheral blood during various conditions such as physiological stress, hypoxia, and inflammation [55,56]. The precise mechanisms of MSC migration to the target tissues are not fully explained. However, it is known that MSCs normally migrate to and mediate repair in damaged tissues. The wound healing function of MSCs starts when the cells migrate towards wounded sites and is triggered by inflammatory signals [57]. The migration of MSCs is modulated by mediators produced and released by other MSCs which affect several receptors and signaling pathways, such as growth factor receptors, G-protein coupled receptor (GPCR), vascular endothelial growth factor/vascular endothelial growth factor receptor (VEGF/VEGFR), stem cell factor-tyrosine kinase receptor (SCF-c-Kit), stromal cell-derived factor-1 (SDF-1)/CXC chemokine receptor-4 (CXCR4), hepatocyte growth factor (HGF/c-Met), platelet-derived growth factor/platelet-derived growth factor receptor (PDGF/PDGFR), monocyte chemoattractant protein-1/CC chemokine receptor 2 (MCP-1/CCR2), and high mobility group box 1/receptor of advanced glycation end products (HMGB1/RAGE) [58,59]. SDF-1 and its receptor, CXCR4, are the main mediators of stem cell recruitment to a tumor microenvironment. Studies that inhibited the function of either SDF-1 or CXCR4 have indicated a crucial role of these molecules in the migration of stem cells [60,61,62]. CXCR4 and SDF-1 blockades in animal models culminates in a remarkable decrease in the migration potential and the migration rate of transplanted stem cells to sites of demyelination, suggesting that the SDF-1/CXCR4 signaling pathway is critical for effective stem cell therapy [62]. Moreover, simultaneous blocking of both CXCR4 and transforming growth factor (TGF)-β receptor demonstrated that CXCR4 is necessary for colonization of MSCs in tumors, differentiation to myofibroblasts, and MSC survival [63,64]. MSCs express several chemokine receptors, including CXCR1, CXCR3, CXCR4, CXCR5, CXCR6, CCR1, CCR2, CCR3, CCR4, CCR5, and CCR9 [65]. Chemokines such as chemokine (C-X-C motif) ligand (CXCL) 12, CXCL13, CXCL16, and their corresponding receptors, are involved in the bidirectional migration of MSCs to the BM where they can create a BM niche and subsequently migrate from the BM into the systemic circulation. On the other hand, various other specific chemokines and their receptors participate in the unidirectional migration of MSCs. CXCL16 plays a critical role in the homing of MSCs to the BM, while chemokine (C-C motif) ligand (CCL) 22 has the most powerful chemotactic influence in terms of the release and movement of MSCs from the bone marrow into the circulation [66,67].

Cell receptor transactivation seems to play a role in migration and is also involved in physiological processes like apoptosis. However, impaired cell receptor transactivation predisposes human cells to pathological conditions. Transactivation of several growth factor receptors, such as epidermal growth factor receptor (EGFR), by GPCRs has been reported to be involved in multiple cellular activities, including responsiveness to cytokines and growth factors [68,69,70,71]. Studies have suggested that the mechanism of receptor transactivation is mediated by the stimulation of membrane-tethered growth factors, such as EGFR, by interacting with GPCRs like CXCR4 [72]. EGFR transactivation is associated with the production of matrix metalloproteinases (MMPs), such as MMP-2 and MMP-9 [73,74], which are enzymes with proteinase activity and are needed to process proteins, such as growth factors, cytokines, and adhesion molecules. MMP-1 is required for the migration of MSCs through bone marrow endothelium [75,76,77]. Increased levels of MMP-2 are responsible for C1q complement-mediated migration of UC-MSCs into the site of injury [78]. MSCs can migrate to tissue sites that have previously been irradiated. Local irradiation was shown to increase the specificity of MSCs for migration and implantation [79]. Based upon all these data, the available evidence supports the rationale for developing new therapeutic approaches that involve MSCs.

## 5. Immunomodulatory Properties of MSCs

**Figure 1:**
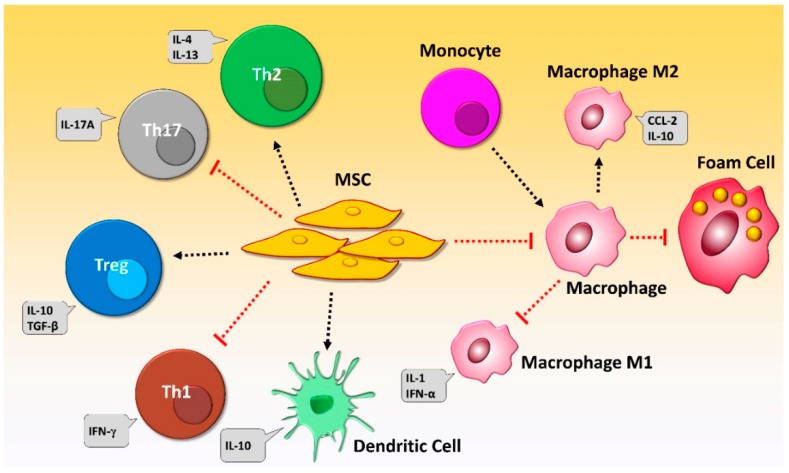
Schematic illustration of anti-inflammatory and immunomodulatory characteristics of mesenchymal stem cells (MSCs) in regulating immune cells. MSCs can suppress the differentiation of Th1 and Th17 cells and the secretion of cytokines specific to these cells. MSCs induce the development of Th2 and regulatory T (Treg) cells, which in turn produce immunosuppressive mediators. MSCs orchestrate the balance between the development of M1 and M2 macrophages. M1 macrophages usually produce immunostimulatory cytokines, while the major cytokine profile of M2 macrophages has immunosuppressive properties. Type 2 dendritic cells (DCs) are developed under the impression of MSCs and produce IL-10. MSCs prevent the transformation of macrophages into foam cells in atherosclerosis. Foam cells are low-density lipoprotein (LDL)-laden macrophages contributing to atherosclerosis through the formation of plaques. The foam cell formation is a consequence of the disruption of balance between cholesterol uptake and cholesterol efflux in macrophages present at intima. MSCs, however, have the potential to prohibit foam cell formation via the reduction of cholesterol uptake and up-regulation of IL-10, eliminating a risk factor for atherosclerosis development.

The immunosuppressive properties of MSCs have been confirmed by a number of studies (Figure 1). Since MSCs can change the behavior of T cells, they have been tested for treating severe graft versus host disease (GVHD) [80]. For example, CD4+ and CD8+ T cells are prevented from proliferation and activation by MSCs [81,82,83]. Most of the immunomodulatory activity of MSCs has been attributed to the enzyme indoleamine-pyrrole-2-3-dioxygenase (IDO) [84]. By regulating tryptophan depletion and the accumulation of mediators like kynurenines, IDO can inhibit the proliferation of immune cells [85,86]. MSCs have been shown to stop the cell cycle of B cells in the G0/G1 phase as well as to reduce the chemotactic activity of B cells. MSCs can also prevent dendritic cells (DCs) from maturation, leading to a decreased presentation of antigens and costimulatory molecules required for T cell activation [87]. MSCs can also reduce the expression of activating receptors on natural killer (NK) cells, including NKG2D, NKp30, and NKp44 [88,89]. It has also been shown that MSCs modulate the cytokine secretion profile of immune cells, such as T cells, DCs, and NK cells [90]. MSCs can also decrease interferon (IFN)-γ secretion from Th1 cells and NK cells, they can decrease the secretion of tumor necrosis factor (TNF)-α from DCs type 1 (DC1), increase the secretion of IL-4 from Th2 cells, and increase IL-10 secretion from DC2. In vitro studies have indicated that MSCs can inhibit the differentiation of naive CD4+ T cells into Th17 cells. Therefore, MSCs can also regulate the production of IL-17, IL-22, TNF-α, and IFN-γ [91]. BM-MSCs have been reported to modulate T and B cells by reducing the production of proinflammatory cytokines, such as TNF-α, IFN-γ, and IL-2 [92]. TNF-α-stimulated gene-6 (TSG-6), a strong anti-inflammatory mediator, is also secreted by MSCs [93]. MSCs also express toll-like receptors (TLRs), including TLR3 and TLR4, which are involved in the regulation of their anti-inflammatory functions [94].

## 6. Pathophysiology of Atherosclerosis and the Role of Inflammation

Atherosclerosis is an inflammatory arterial disease which is initiated by dyslipidemia, arterial hypertension, smoking, obesity, and other risk factors [95,96,97]. Each step of the atherosclerotic process, from the beginning of the plaque development to its rupture, is accompanied by inflammation [98,99]. During the initial events of atherosclerosis, ox-LDLs are trapped in the vessel wall, resulting in the dysfunction of the endothelial cells [100,101]. Later, adhesion molecules, for example, selectins and integrins, are overexpressed on leukocytes, culminating in increased adhesion, rolling, and migration of inflammatory cells into the subendothelial region of the arterial wall [102,103,104]. The next step in the atherogenic cascade is the increased infiltration of T cells, monocytes, and neutrophils across the vessel wall through interendothelial junctions. In the subendothelial region, macrophages ingest lipoproteins and are overstuffed with lipids, and are then transformed into foam cells, which then produce several inflammatory mediators [105,106]. When immune cells and lipid molecules accumulate in the intima, the early phase of plaque develops, which is called fatty streak. Endothelial injury progresses and may be accompanied by endothelial progenitor cell homing in an attempt to repair the injured endothelium. Foam cells and extracellular lipids form a core of the developing plaque, which is then covered with a cap of collagen-rich matrix and smooth muscle cells [8,107]. By the engagement of TLRs on the macrophages, these cells are activated and inflammatory cytokines and mediators are subsequently produced and released. The activation of CD4+ T cells plays a significant role in the development of atherosclerosis. The depletion of T cells causes a reduction of the atherosclerotic lesion size [108]. Th1 is the most prevalent subset of T cells in atherosclerotic lesions and is involved in the production of inflammatory mediators like IFN-γ [109,110]. On the other side, IFN-γ modulates inflammation in the vessel wall by promoting the activation of antigen-presenting cells, decreasing the production of collagen by modified smooth muscle cells, upregulating lipid ingestion by macrophages, and upregulating adhesion molecules on ECs. These events are followed by the infiltration of inflammatory immune cells from blood to the lesions [111]. The persistent infiltration of leukocytes to the atherosclerotic lesion sites maintains a local state of low-grade inflammation.

Because of the critical function of inflammation in the initiation and perpetuation of atherosclerosis, the engraftment of MSCs, which have the potential to regulate and control inflammation, has been extensively investigated as a potential therapeutic tool for treating atherosclerosis. Allogeneic MSCs have important characteristics, which include the suppression of T cell proliferation and suppression of the immune response elicited from T cells. This suggests that transplantation of allogeneic MSCs may represent a beneficial therapeutic approach for atherosclerosis [112,113]. Many investigations on animal models have documented that MSCs can act against atherosclerosis. In most of these animal models, atherosclerosis was induced by a high-fat diet in either apolipoprotein E (ApoE) or low-density lipoprotein receptor (LDLR) knockout mice. In most of these animal studies, MSCs were obtained from bone marrow. Nevertheless, UC-MSCs and skin-derived MSCs (S-MSCs) can also be used for analyzing their possible atheroprotective effects.

## 7. Modulation of Inflammatory Mediators by MSCs during Atherosclerosis

Many studies have confirmed that the protective properties of MSCs on atherosclerotic lesions are based predominantly upon their secretion of various anti-inflammatory mediators [114]. Transplantation of BM-MSCs in the atherosclerotic lesions of various animals (Table 1) resulted in an overproduction of anti-inflammatory cytokines, including IL-10 and TGF-β1, while the production of pro-inflammatory cytokines, such as IL-1β, IL-6, and TNF-α, were reduced [115]. TGF-β1 secretion by MSCs causes the induction of CD4^+^CD25^+^Foxp3^+^ regulatory T (Treg) cells [116] and also suppresses the proliferation of NK cells [117]. Additionally, engraftment of MSCs causes a reduction in the serum levels of CCL2, which is a chemokine that plays a role in the activation and recruitment of mononuclear cells [118]. MSC also suppress the differentiation of T cells. S-MSC therapy, both in vivo and in vitro, is also involved in the attenuation of inflammation by inhibiting the production and release of TNF-α and stimulating IL-10 release [119]. MSCs can also inhibit the expression and function of NF-κB [120,121,122]. In fact, there is also evidence demonstrating the downregulation of NF-κB in atherosclerotic lesions after MSC engraftment [123].

MSCs also produce and release immunomodulatory mediators, such as prostaglandin E2 (PGE2), IDO, and TSG-6 [127]. Differentiation of Th17 cells, which produce inflammatory cytokines, is inhibited by IDO through tryptophan depletion [86]. IDO also reduces the proliferation and cytotoxic activity of NK cells, as well as prevents the maturation, activation, and function of DCs [128]. It has been reported that PGE2 promotes Treg cell development, reduces T cell proliferation, and stimulates the production of IL-4 and IL-10 [129]. Cells and tissues normally do not express TSG-6. However, TSG-6 is upregulated when cells are stimulated by pro-inflammatory cytokines such as IL-1, IL-6, and TNF-α [130,131]. TSG-6 is involved in a feedback loop or mechanism to prevent the remodeling of extracellular matrix by a reduction in the expression of inflammatory mediators, suppression of neutrophil recruitment, and inhibition of the activity of plasmin [132,133]. A recently published study demonstrated that MSCs-exosomes ameliorated atherosclerosis in ApoE^−/−^ mice and promoted M2 macrophage polarization in the atherosclerotic plaque through the miR-let7/HMGA2/NF-κB pathway and that they suppressed macrophage infiltration via the miR-let7/IGF2BP1/PTEN pathway in the atherosclerotic plaque. The conclusion was that MSCs-exosomes can affect inflammation in atherosclerotic plaque [129]. Recently, it has been shown that human amnion mesenchymal stem cells (hAMSCs), which are a particular population of MSC and have immunomodulatory abilities, suppressed the phosphorylation of p65 and inhibitor of κB-α, suggesting that the NF-κB pathway was involved in the hAMSCs-mediated suppression of the immune response [130,131].

## 8. Prospects for MSC-Based Therapy of Atherosclerosis

### 8.1. MSCs and the Improvement of Endothelial Function during Atherosclerosis

Endothelial dysfunction is considered to be the initial step in the development of atherosclerosis, which promotes the accumulation of lipid droplets, the infiltration of macrophages, the development of foam cells, and, subsequently, the recruitment of platelets and T cells [134]. The endothelium is a complicated organ with both endocrine and paracrine characteristics that are involved in the control of vascular homeostasis. Vascular nitric oxide (NO) is produced by endothelial nitric oxide synthase (eNOS) [135,136]. As a vasodilator, NO enhances the level of cyclic guanosine monophosphate (cAMP) in smooth muscle cells and at the same time, suppresses the adhesion of leukocytes, limits platelet aggregation, and prevents the proliferation of smooth muscle cells. Therefore, NO has anti-inflammatory properties by restricting the infiltration of leukocytes across the vessel wall [137]. In spite of the ability of ECs to self-repair in the presence of inflammatory stimuli, endothelial repair could be further stimulated by MSCs. It has been observed that amnion-derived MSCs (A-MSCs) were able to increase the survival of ECs in vitro by downregulating the level of lactate dehydrogenase and contributing to the maintenance of the endothelial network [138]. Engraftment of allogeneic BM-MSCs was reported to ameliorate atherosclerosis by repairing the injured endothelium [125]. NO production from ECs is reduced by ox-LDL via the deactivation of the Akt/eNOS function and stimulation of eNOS cleavage [139]. However, when MSCs were cocultured with endothelial cells, ox-LDL did not have the same effects on these cells [140]. It appears that the beneficial influence of MSCs on endothelial cells (via the activation of the Akt/eNOS pathway) is mediated by an increased production of IL-8 and macrophage inflammatory protein (MIP)-2. This observation was confirmed when neutralization antibodies against IL-8 or MIP-2 were evaluated, which blocked the effects of MSCs on ox-LDL-exposed ECs [125]. It has been shown recently that differentiation of BM MSCs into endothelial cells in vitro might have beneficial effects and could have an application in the treatment of atherosclerotic lesions [125]. Skin-derived MSCs have also been used in experiments trying to find a treatment for atherosclerosis [119]. Therefore, by improving the physiological function of endothelial cells, MSC transplantation could slow down the development of atherosclerosis [125,141].

### 8.2. MSCs and Regulatory T Cell Development during Atherosclerosis

Regulatory T cells demonstrate immunosuppressive behavior and modulate the quality and quantity of the immune response by either direct contact with other cells or by secretion of immunoregulatory mediators such as IL-10 and TGF-β [142]. Tregs are CD4^+^CD25^+^ T cells that express the forkhead box transcription factor (FOXP3), which is the specific transcription factor that modulates the development and function of Treg cells [143,144]. Studies have shown that the number of Treg cells is decreased in atherosclerotic plaques [145,146]. Moreover, when FOXP3 is knocked down, the progression of atherosclerosis occurs in various animal models [147], suggesting a possible protective function of Treg cells against atherosclerosis. By suppressing the function of DCs and Th1/Th2 cells, Treg cells have atheroprotective activity in mice with immunodeficiency and hyperlipidemia [148]. In accordance with this, it has been shown that Treg cells produce IL-10 and TGF-β in the atherosclerotic milieu and, therefore, inhibit the functions of DCs and Th1/Th2 cells [149,150,151,152]. Tregs have also been reported to be able to suppress the expression of MMP-2 and MMP-9, which are important enzymes that degrade the extracellular matrix and promote the instability of atherosclerotic lesions [153].

Although the application of Treg cells would appear to be promising in the treatment of atherosclerosis, there are some challenges. Among the most limiting factors is the difficulty in the efficient separation of Treg’s to achieve a pure or homogeneous population of these cells. To resolve this problem, MSCs have been suggested to be an adequate alternative cell source. The application of MSCs could be achieved by promoting the recruitment and development of Treg cells from an individual patient [124,127]. When MSCs were co-cultured with splenocytes, the number and function of Treg cells were enhanced. Moreover, MSCs have upregulated both the messenger RNA (mRNA) and protein expression of FOXP3 in an atherosclerotic animal model [115]. A number of mechanisms have been proposed regarding the capacity of MSCs to modulate the proliferation and activation of Treg cells. MSCs also secrete high levels of TGF-β1 to enhance the differentiation of Treg cells [154]. Another direct cell–cell contact mechanism mediated by MSCs is TLR-mediated stimulation of Treg cells, which is facilitated by upregulating the levels of the Notch ligand, Delta-like 1 [94]. MSCs induce Treg cells via notch ligand Jagged-1 as well [155]. Monocytes are also important for MSC-induced development of Treg cells. In fact, MSCs contribute to the differentiation of monocytes to type 2 macrophages, which have anti-inflammatory characteristics and promote Treg cell generation by the production of CCL18 [154].

### 8.3. Recruitment of MSCs into Atherosclerotic Plaques

The commitment of MSCs into effector cells facilitates their capacity to repair injured tissues. S-MSCs can locate atherosclerotic plaques and are specifically homed closely to macrophages. Carboxyfluorescein succinimidyl ester-labeled mouse MSCs, 7 days after an injection, reside in regions near macrophages in the endothelium affected by atherosclerotic lesions [119,125]. Transplanted BM-MSCs reside in regions of ruptured plaques and then differentiate into collagen fibers and ECs [124]. After four weeks of MSC engraftment, 5-bromo-2′-deoxyuridine (BrdU)-labeled BM-MSCs are found in the injured areas of the endothelium. Several mediators and receptors are involved in regulating the migration and differentiation of stem cells to the site of vascular injury, for example, β1- and β2-integrins, α4 integrin, P-selectin glycoprotein ligand-1, and CXC chemokine receptor-2 and -4 (CXCR2 and CXCR4) [156,157]. During vascular repair, the direct interaction between progenitor cells and platelets enhances MSC activation, adhesion, chemotaxis, and their possibility to transform into mature ECs [158]. It has to be stressed that more recent studies indicate that a very limited number of MSCs are retained after treatment. It seems that the beneficial effect of MSCs appears to be predominantly due to paracrine signaling mechanisms, including by exosomes transporting various proteins and non-coding RNAs such as micro-RNAs and lncRNAs [154].

Macrophages are the major players in atherosclerosis during foam cell formation and are responsible for plaque stability. It has been shown that MSCs can modulate macrophages, for instance they can reprogram this type of cell into anti-inflammatory phenotypes, M2 macrophages [159,160]. However, it is still not clear whether the macrophage phagocytic capacity is weaker or stronger when co-cultured with MSCs, and the signaling pathways by which MSCs modulate macrophage needs further research.

### 8.4. The Role of MSCs in Modulating Lipid Levels

Dyslipidemia is the main risk factor for the onset and progression of atherosclerosis [161]. The indirect effect of MSCs on cholesterol metabolism via immune modulation has been confirmed. A relationship between cholesterol metabolism and immune cells has also been described. In both LDLR^−/−^ mice on a high-fat diet and ApoE^−/−^ mice on a normal chow diet, T and B cell deficiency decreased serum lipoproteins, particularly the apoB-rich lipoproteins [162]. Following 5 weeks of treatment with MSCs in mice, the inflammatory state was modulated and serum cholesterol levels were considerably reduced due to a reduction in very low density lipoprotein (VLDL) levels [118]. A significant reduction of lipoprotein lipase was observed in the liver of MSC-treated mice, which decreases VLDL katabolism by decreasing the breakdown of triglycerides into free fatty acids, which results in less availability of free fatty acids for VLDL particles synthesis by hepatocytes. Furthermore, decreased activation of Kupffer cells indirectly affects VLDL metabolism in MSC-treated mice because in physiological conditions, Kupffer cells, by releasing different mediators, promote secretion of VLDL from hepatocytes [163]. Additionally, lipoprotein lipase insufficiency in Kupffer cells attenuates their uptake of ox-LDL or VLDL, thus acting antiatherosclerotic [159,160]. All these data suggest that MSCs can decrease plasma VLDL levels. Interestingly, it has been shown that TNF-α, which is downregulated in splenocyte and MSC co-cultures, upregulates sterol response element binding protein -1c (SREBP-1c), which promotes the generation of VLDL particles [164]. In contrast, in LDLR^−/−^ mice, IL-10 upregulation decreases serum cholesterol levels, primarily because of decreased VLDL levels [165]. It has been shown that induced pluripotent stem cells-derived MSCs (iPSC-MSCs/iMSCs) have longer survival times, stronger proliferative capacity and are more stable than bone marrow-MSCs, and that they can also, apart from decreasing serum cholesterol and inflammatory response, decrease the expression of Notch 1. Notch1 is a receptor in the Notch signaling pathway, which is a highly conserved pathway that is associated with many cellular processes, such as differentiation, proliferation, and apoptosis [166]. It is important to know which type of MSCs might be the best to prevent atherosclerosis when macrophages are exposed to atherogenic ox-LDL. It has been shown that adipose tissue MSCs co-cultured with M1 foam macrophages when treated with ox-LDL would cause similar or improved anti-inflammatory effects when compared with BM-MSCs [167].

### 8.5. Stability of Atherosclerotic Plaque and MSCs

Depending on the internal environment, atherosclerotic plaques can be stable or unstable. It has been suggested that plaque regions with a large lipid core, a large number of macrophages, and a thin fibrous cap are more susceptible to rupture, but matrix-degrading proteases from foam cells induced by inflammatory cells as well as immature neovessels sprouting into the atherosclerotic lesion are also very important for plaque stability [168]. Rupture of the atherosclerotic plaque significantly increases the risk of myocardial infarction, ischemic stroke, and acute coronary syndrome [169,170]. An increasing body of clinical evidence demonstrates that vulnerability, rather than the plaque size, is closely associated with cardiovascular prognosis [171,172]. The ability of allogeneic MSCs in repairing ruptured lesions has been intensively investigated. It appears that MSCs can promote collagen fiber synthesis and regeneration of the inner endothelial layer of the vessel wall, suggesting their significance in treating the advanced atherosclerotic lesions [124].

C-reactive protein (CRP) is an important predictive marker of plaque instability. CRP stimulates chemotaxis of several adhesion molecules to endothelial cells. Moreover, CRP stimulates macrophages to synthesize and release pro-coagulant and pro-inflammatory factors, exacerbating the inflammatory state [173,174]. It should also be mentioned that plasminogen activator inhibitor-1 (PAI-1), which is primarily involved in fibrinolysis, is an important risk factor for thrombotic disorders and is one of the several biomarkers of tissue injury [175]. MSC engraftment significantly reduced the expression of the key biomarkers of tissue injury, such as PAI-1, CRP, and MMPs, in an atherosclerosis rabbit model [124]. TNF-α directly influences plaque stability and stimulates cell necrosis and thrombosis by recruitment and accumulation of inflammatory cells in atherosclerotic regions [176]. On the other hand, IL-10, as an anti-inflammatory factor, induces the proliferation of smooth muscle cells and inhibits inflammatory cell accumulation, thereby promoting atherosclerotic lesion stability [177,178]. In an atherosclerotic rabbit model, MSC transplantation stabilized vulnerable plaques via the reduction of IL-6 and TNF-α and an increase of IL-10. [123]. MSC engraftment downregulated the expression of MMP-9, MMP-1, and MMP-2 in atherosclerotic lesions. These data suggest that MSCs, by reducing the MMP production, could modify plaque vulnerability and decrease local collagen degradation.

Another factor which is involved in atherosclerotic plaque formation is cell apoptosis [179,180]. It has previously been established that the apoptosis of macrophages, vascular ECs, and vascular smooth muscle cells is involved in the generation, progression, and rupture of atherosclerotic lesions [181]. Interestingly, MSC engraftment considerably reduces the number of apoptotic cells in atherosclerotic lesions, suggesting that MSCs can further promote plaque stability and decrease the risk of atherosclerosis [123]. Arterial hypertension is not only a cardiovascular disease (CVD) risk factor, but can also influence plaque stability. It has been shown that skin-MSCs attenuated angiotensin II-induced hypertension by inhibiting Th17 cell differentiation and by modulating macrophage M2 polarization. This suggests that skin-MSCs do not only suppress the formation of the atherosclerosis, but potentially also have a role in MSC based therapy for hypertension [182].

## 9. Drawbacks of MSCs Therapy in Atherosclerosis

The safety of MSC engraftment in several preclinical and clinical trials has been shown in small pilot studies on humans, but not those with atherosclerotic lesions [183,184,185]. However, the exact dose range of MSCs for therapeutic use in atherosclerosis has not been well characterized. Nonetheless, an optimal dose of MSCs for engrafting has been suggested and ranges from 1 to 5 × 10^6^ MSCs per 1 kg of body weight [186]. In cancer patients, an optimal dose of MSCs has been suggested—for example, 1 × 10^6^ MSCs per 1 kg of body weight in breast carcinoma—and it appears to be well-tolerated [187]. It has to be stressed that a lot is still not known regarding time-dependent effects of using MSCs to treat atherosclerosis. It is not clear what the short- and long-term effects of using MSCs are. Nevertheless, in animal models, allogeneic MSCs, despite having low immunogenicity, can be rejected by the recipient animal [36]. One of the problems with this therapeutic approach to atherosclerotic lesions is that any MSC-based therapy would surely have systemic effects. Furthermore, while early phase clinical and preclinical studies have not yet identified any potential complications of MSC transplantation in humans, tumor development has increasingly been documented in several rodent models. Chromosomal instability has been found in mouse BM-MSCs, which may act as a trigger for malignant transformation [188]. Also, BM-MSC transplantation may increase the risk of gastric cancer [189]. In recent years, a general consensus has been reached that improved manufacturing conditions, including cell preparation, isolation, culture, and manipulation, can considerably decrease the tumorigenicity of transplanted MSCs. In fact, the duration of cell harvesting, as well as cell culture conditions, significantly influence the occurrence of malignancy as an adverse effect [190]. When discussing atherosclerosis and MSCs, some experiments on animal models proved that MSC can cause calcification and even injure abdominal aorta after bone marrow-MSC administration [191]. There is a number of other potential adverse effects when using MSCs in atherosclerosis treatment [192]. Therefore, further efforts are needed to evaluate the safety of MSC transplantation in the context of atherosclerosis therapy.

## 10. Investigation of MSC Potential to Treat Atherosclerosis in the Clinical Setting

Clinical trials evaluating the therapeutic potential of MSCs from diverse sources have mainly been focused on the assessment of the safety and efficacy of recruiting such cells to treat and/or alleviate peripheral arterial disease (PAD) secondary to critical limb ischemia (CLI) and diabetes. The majority of these studies are still in progress and only a small number of these trials are reported to be completed; however, no results have been released to date. In the following, we provide some details regarding the relevant clinical investigations initiated during the last 10 years.

The first clinical trial in this context was started in 2010 including culturing MSCs in the presence of gold nanoparticles with silica-iron oxide shells and infusion of nanoparticle-bearing cells into atherosclerotic lesions. The investigation aimed to compare the use of gold nanoparticles with iron oxide-silica shells with stenting as measures to be taken to circumvent atherosclerosis (NCT01436123). Although the study was terminated, no results are available.

Another study in 2011 recruiting 25 participants with CLI and PAD aimed to investigate the MSC ability to treat CLI with autologous MSC administration (NCT01351610). The human BM-CD34-negative MSCs were intravenously infused and their tolerability and efficacy by the patients were studied.

A trial research was started in 2012 to determine the safety and possibility of three different doses of mesenchymal-like stem cells called endometrial regenerative cells (ERCs)/intramuscularly derived menstrual mesenchymal stem cells on 15 CLI patients. The participants are not eligible for surgical or catheter-based interventions for revascularization. The study includes 10 injections of 2.5, 5, or 10 million MSCs (a total of 25, 50, or 100 million ERC) to the gastrocnemius muscle above the failed vascular perfusion area (NCT01558908).

An interventional phase I and II clinical trial initiated in 2014 aims to evaluate the safety and efficacy of UC-MSC injection to alleviate PAD complications in 30 diabetic patients. The participants were divided into three groups to receive different doses of UC-MSCs, followed by another injection of the same UC-MSCs dose 4 and 8 weeks after the first infusion (NCT02287831).

In 2015, a phase I clinical trial for analyzing MSC potential in ameliorating diabetic foot ulcers was designed (NCT02796079). Using autologous BM-MSCs, the investigators evaluate the therapeutic application of MSC against diabetes-related vascular complications.

Another similar clinical study was established in 2016 to assess the safety of UC-MSC infusion in around 240 diabetic patients suffering from diabetes-related vascular complications (NCT02834858).

Recruiting 240 diabetic patients with peripheral vascular disease, ischemia, and diabetic foot, a clinical assessment was performed using adipose-derived MSCs for the evaluation of MSC potential in healing foot ulcers that resulted from diabetes (NCT02831075).

A phase I clinical trial was recruited in 2016 to investigate the preventive effects of allogenic BM-MSC intra-muscular administration in areas near the amputation region from wound ischemia and revision incidence following amputation surgery. The investigation includes 16 patients with atherosclerotic limb ischemia undergoing semi-elective lower extremity major amputation (NCT02685098).

An interventional phase II and III clinical trial has been running since 2017 and is aimed to assess the efficacy, effectiveness, and safety of exposure to allogeneic BM-MSC for angiogenesis and neovascularization in 60 patients with no-option severe limb ischemia (SLI) (NCT03042572). The patients receive 30 injections of 5 × 10^6^ BM-MSCs (a total of 150 × 10^6^ BM-MSCs) in 30 different injection sites in the lower leg of the ischemic extremity.

A 2018-initiated clinical trial was conducted to understand the safety and efficacy of allogenic ABCB5-positive MSCs at a dose of 150–225 × 10^6^ cells (being administered via 20–30 intramuscular injections) in treating peripheral arterial occlusive disease (PAOD) (NCT03339973).

A very recent phase II study has commenced on CLI and PAD patients who will be exposed to autologous adipose-derived stem/stroma cell (ASC) through intramuscular injection (NCT03968198).

## 11. Conclusions and Future Challenges and Perspectives

MSCs have displayed remarkable beneficial characteristics, which could make them suitable to being used for several therapeutic purposes, among others, in reducing atherosclerosis. Some of the advantages of MSCs are the production of mediators which can attenuate inflammation, the potential to migrate to sites of arterial injury, and the ability to respond proportionally to the size of the tissue injury. An important issue concerning the role of MSCs in atherosclerosis treatment is probably the mechanobiology of MSCs. Considering the hemodynamic nature of the vasculature and the important role that fluid forces play in the progression of atherosclerosis, the effect of different mechanical stresses on MSCs could be important [193]. Mechanobiology plays a major role in transducing physical forces into biochemical modifications that promote different MSCs differentiation pathways and it is well known that the stiffness of the extracellular matrix (ECM) surrounding MSCs influences the path of differentiation [194]. Since the type of ECM modulates MSC mechanotransduction, it has recently been shown that Yes-associated protein (YAP) translocation probably plays a role in this process [195].

The mediators produced and released by MSCs in atherosclerotic lesions contribute to the modulation of inflammation and can improve the function of the damaged endothelium. MSC engraftment might provide a novel strategy for the improvement of atherosclerotic lesions and for the prevention of plaque rupture. A considerable number of experimental in vitro and animal studies support the idea of a possible clinical application of MSC-based therapy. Therefore, in order to achieve sustained and long-term beneficial effects of MSCs, additional therapeutic treatment modalities are required. Based on current evidence, further investigations of MSCs are needed to better understand the methods required to harvest larger numbers of these cells, as well as techniques of modifying their biological functions/properties in vitro in order to obtain more effective MSCs. In the near future, engineering of MSCs to selectively deliver immunosuppressive and anti-angiogenic agents which would decrease the development of atherosclerotic lesions might be an additional therapeutic solution, however the role of MSCs in general should be the subject of future investigations because many questions still need to be answered. One of the biggest challenges is still how to translate the results of in vitro and animal studies with MSCs into studies on humans with atherosclerotic plaques. Another challenge is how to target the specific pathways involved in atherogenesis with MSCs and retain MSCs at the sites of action, i.e., atherosclerotic lesions, without affecting other areas and other mechanisms.

## Figures and Tables

**Table 1:** Animal studies exploring the therapeutic potential of mesenchymal stem cells in the treatment of atherosclerosis.

Animal Model	Cell Source	Administration Route	Consequence	Reference
New Zealand rabbits	Bone marrow	Intravenous	Increased: TSG-6, IL-10, hs-CRP, TNF-α, IL-6, NF-κB Decreased: Apoptosis, MMPs	[123]
New Zealand rabbits	Bone marrow	Intra-arterial	Increased: Collagen fibers Decreased: MMPs, PAI-1, hs-CRP	[124]
ApoE^−/−^ mice	Bone marrow	Intravenous	Increased: Tregs Decreased: SRA, CD36	[115]
ApoE^−/−^ mice	Bone marrow	Intravenous	Increased: eNOS, IL8, MIP-2	[125]
ApoE^−/−^ mice	Skin	Intravenous	Increased: IL-10, PGE2 Decreased: NF-κB, TNF-α	[119]
LDLR^−/−^ mice	Bone marrow	Intravenous	Increased: Tregs Decreased: CD4+ T cells, CCL2, IFN-γ, monocytes, TNF-α, serum cholesterol	[118]
Albino rats	Cord blood	Intravenous	Increased: iNOS	[126]

TSG-6: TNF-α-stimulated gene-6; IL-10: interleukin-10; hs-CRP: high-sensitivity C-reactive protein; TNF-α: tumor necrosis factor-α; IL-6: interleukin-10; iNOS: inducible nitric oxide synthase; SRA: class A scavenger receptor; eNOS: endothelial nitric oxide synthase; PGE2: prostaglandin E2; IL-8: interleukin-8; MIP-2: macrophage inflammatory protein 2; CCL2: chemokine (C-C motif) ligand 2; IFN-γ: interferon-γ; Treg: regulatory T cell; MMP: matrix metalloproteinase; PAI-1: plasminogen activator inhibitor-1.

## Abbreviations

MSC	mesenchymal stem cell
EC	endothelial cell
ox-LDL	oxidized low-density lipoprotein
PRRs	pattern recognition receptors
IL	interleukin
BM-MSC	bone marrow-derived mesenchymal stem cell
UC-MSC	umbilical cord—derived mesenchymal stem cell
GPCR	G-protein coupled receptor
VEGF/VEGFR	vascular endothelial growth factor/vascular endothelial growth factor receptor
SCF-c-Kit	stem cell factor—tyrosine kinase receptor
SDF-1	stromal cell-derived factor-1
CXCR4	CXC chemokine receptor-4
HGF/c-Met	hepatocyte growth factor
PDGF/PDGFR	platelet-derived growth factor/platelet-derived growth factor receptor
MCP-1/CCR2	monocyte chemoattractant protein-1/CC chemokine receptor 2
HMGB1/RAGE	high mobility group box 1/receptor of advanced glycation end products
TGF-β	transforming growth factor-β
CXCL	chemokine (C-X-C motif) ligand
CCL	chemokine (C-C motif) ligand
EGFR	epidermal growth factor receptor
MMP	matrix metalloproteinase
GVHD	graft versus host disease
IDO	indoleamine-pyrrole-2-3-dioxygenase
DC	dendritic cell
NK	natural killer
IFN	interferon
TNF-α	tumor necrosis factor α
DC1	dendritic cell type 1
TSG-6	TNF-α-stimulated gene-6
TLR	toll-like receptor
ApoE	apolipoprotein E
LDLR	low-density lipoprotein receptor
S-MSC	skin-derived MSC
Treg cell	regulatory T cell
PGE2	prostaglandin E2
hAMSC	human amnion mesenchymal stem cell
NO	nitric oxide
eNOS	endothelial nitric oxide synthase
cAMP	cyclic guanosine monophosphate
A-MSC	amnion-derived mesenchymal stem cell
MIP-2	macrophage inflammatory protein 2
FOXP3	forkhead box transcription factor
mRNA	messenger RNA
BrdU	5-bromo-2′-deoxyuridine
VLDL	very low density lipoprotein
iPSC-MSC/iMSC	induced pluripotent stem cells- derived mesenchymal stem cell
SREBP-1c	sterol response element binding protein-1c
CRP	C-reactive protein
PAI-1	plasminogen activator inhibitor-1
CVD	cardiovascular disease
PAD	peripheral arterial disease
CLI	critical limb ischemia
ERC	endometrial regenerative cell
SLI	severe limb ischemia
PAOD	peripheral arterial occlusive disease
ASC	adipose-derived stem/stroma cell
ECM	extracellular matrix
YAP	Yes-associated protein
hs-CRP	high-sensitivity C-reactive protein
iNOS	inducible nitric oxide synthase
SRA	class A scavenger receptor
